# Formative content development and evaluation of a text message intervention for excessive alcohol consumption among university students.

**DOI:** 10.1186/1940-0640-10-S2-P4

**Published:** 2015-09-24

**Authors:** Preben Bendtsen, Catharina Linderoth, Ulrika Mussener

**Affiliations:** 1 Department of Medical and Health Sciences, Faculty of Health Sciences, Linköping, Sweden

## Background

Previous studies have highlighted the magnitude of risky drinking among Swedish students; finding that at least 50% of the students could be classified as risky drinkers[1]. Human resources are not sufficient to offer all risky drinkers face-to-face brief interventions. Text messaging is the least advanced but also the least expensive eHealth technology that virtual works on all mobile phones[2]. Thus text messaging have strong potential as a tool for healthy life style interventions since it is available on all mobile phone, the cost is low and is widely applicable to other health behaviours[3]. The objectives are to develop a new text message based alcohol intervention using a formative qualitative research approach.

## Material and methods

The content development will be an interactive process in which theory guided content is modelled and qualitatively evaluated by the users in line with guidance on the development of complex intervention. The process will begin with a revisit of the literatures on theory-based approaches to behaviour change across behavioural targets, with focus on alcohol. In the next step the structure and content of tentative text messages will be discussed by students in focus group interviews.

## Results

Three focus group interviews will be conducted during spring 2015 in three regions in Sweden in order to investigate the students reasoning on the theoretical informed content. The students will be asked to formulate new messages and disregard existing messages. Preliminary results from the interviews will be presented.

## Conclusions

The responses of the students will enable us to understand how students think about the structure and content of a text message based alcohol intervention in order to increase uptake, compliance and effectiveness of the intervention.

## Funding declarations

The study is funded by the Swedish Public Health Agency.

## Conflicts of interest

Preben Bendtsen is partly owner of a software company that distributes life style interventions.
